# The effect of endometriosis on fertility in an animal model

**DOI:** 10.25122/jml-2021-0391

**Published:** 2022-09

**Authors:** Dimitrios Kanellopoulos, Dimitra Karagianni, Vasilios Pergialiotis, Nikolaos Nikiteas, Andreas C Lazaris, Dimitrios Iliopoulos

**Affiliations:** 1 Laboratory of Experimental Surgery and Surgical Research N.S. Christeas, National and Kapodistrian University of Athens, Athens, Greece; 2 1^st^ Department of Pathology, National and Kapodistrian University of Athens, Athens, Greece; 3 2^nd^ Propaedeutic Department of Surgery, National and Kapodistrian University of Athens, Athens, Greece

**Keywords:** endometriosis, animal model, rats, infertility

## Abstract

The present experimental model aimed to investigate the possible effect of endometriosis on ovarian function by altering follicular maturation and development. This single-blind, randomized study included twenty-four female Sprague Dawley rats, 2.5 months old, weighing 160–200 grams. The animals were randomly separated into four groups on the day of the surgery. Each group consisted of 6 rats. The first group (A) consisted of healthy female rats (control group). The second group (B) consisted of rats subjected to surgical insertion of ovarian endometrioma. The third group (C) consisted of rats subjected to surgically induced diffuse intraperitoneal endometriosis, and the fourth group (D) consisted of rats subjected to surgically induced extraperitoneal endometriosis. According to our experimental model, endometriosis may affect ovarian function by increasing the number of luteinized unruptured follicles (follicles that have undergone luteinization without prior rupture).

## INTRODUCTION

Endometriosis is a condition that affects the reproductive system of women whose endometrial tissue (stroma and glands) grows outside the uterus. It is a leading cause of morbidity and largely affects the quality of life of women. Despite the increased number of research for treatment and a better understanding of endometriosis, the exact process of the disease development remains unclear. The disease occurs mainly in women of reproductive age and is estimated to affect 4–10% of the general female population [1–3]. According to the international literature, there is a 6 to 7-year delay in the diagnosis of endometriosis, starting with the first symptoms in women of reproductive age [4]. The diagnosis delay is due to the non-specificity of the symptoms of this disease (dyspareunia, ileus, tenesmus, dysmenorrhoea, infertility, and pelvic pain) [1, 3, 5–8]. The prevailing theories that have been developed regarding the pathogenesis of the disease are: a) implantation theory, b) coelomic metaplasia theory and c) lymphatic and vascular metastasis theory [1, 5–7, 9–13].

According to Sampson's implantation theory in 1922, the endometrial foci are caused by the retrograde dispersion of endometrial cells into the peritoneal cavity during menstruation. In 1987, Singer and Jordan found reverse dispersion of cells in 94% of patients, with only 19% who developed endometriosis. The researchers also observed that endometriosis developed in women with tubal obstruction and women subjected to hysterectomy, therefore, implantation theory was not enough to explain all clinical forms of the disease. Another theory is that of Coelomic metaplasia, according to which the ectopic endometrium may result from metaplasia of the peritoneal mesothelial cells. A third theory is that of lymphatic and vascular metastasis, according to which endometriosis may appear in the retroperitoneal area and in tissues that are not in direct contact with the peritoneum [1, 5–7, 9–23].

Up to 50% of women with infertility are finally diagnosed with endometriosis [21]. The correlation is obvious between endometriosis and infertility. The pathogenesis of infertility in women suffering from endometriosis is multifactorial and complex to explain. Although it could be easily understood that advanced and serious endometriosis may cause pelvic disorders, resulting in mechanical infertility, the ways in which the mild form of the disease may influence the ability of a woman to conceive and give birth remain to be understood [13–23]. To investigate the association of endometriosis with infertility, we created an experimental animal model. In this model, we studied the effect of endometriosis on the ovarian tissue of rats.

Endometriosis is a disease with a significant effect on women's quality of life and reproductive ability. Despite the current efforts to treat the disease, such as medicated treatment, which is not enough to treat infertility, many women are forced to undergo surgery. The purpose of the present study was to investigate the effect of endometriosis on the ovarian tissue of rats and, in more detail, if endometriosis can affect ovarian function by altering follicular maturation and development.

## MATERIAL AND METHODS

The study was performed in the Laboratory for Experimental Surgery and Surgical Research N.S. Christeas, from 9 to 13/03/2019. Twenty-four female Sprague-Dawley rats, 2.5 months old, weighing 160–200 grams, provided by the laboratory for experimental surgery and surgical research N.S. Christeas were used. Living and handling conditions were adequate with the presidential decree 160/91 governing the protection of animals used for research purposes. Twelve hours before the surgery, all rats were subjected to fasting. On the day of the surgery, the animals were randomly separated into four groups. Each group consisted of 6 rats. The first group (A) consisted of healthy female rats (control group). The second group (B) consisted of rats with surgical insertion of ovarian endometrioma. The third group (C) consisted of rats with induced diffuse intraperitoneal endometriosis, and the fourth group (D) consisted of rats with extraperitoneal endometriosis [24–31].

The experimental induction of ovarian endometriosis (Group B) involved the administration of anesthesia by intramuscular ketamine injection at a dose of 60 mg/kg and IM xylazine injection at a dose of 7 mg/kg. Before the surgical operation, abdominal shaving was performed, and surgical field antisepsis, using a 10% povidone-iodine solution. A midline surgical incision and opening of the abdominal wall followed, and the uterine horn was found, a part of which was removed and auto transplanted into the peritoneal cavity. Thus, endometriosis was surgically induced through the implantation of autologous parts of endometrial tissue in the peritoneal cavity. All interventions were performed in aseptic conditions. Initially, the rats were placed in a supine position. A midline incision through the skin of 3–5 cm was performed. Then two uterine horns were found. The peripheral part of the right horn, 1 cm long, was ligated at the level of the uterotubal junction; then, an incision was made at the proximal end of the uterine horn. The part that was removed was kept in a sterile recipient containing NaCl 0.9% at 37°C. Then a surgeon scalpel was used to open the left endometrial horn, separated into two sections of 4×4 mm each. The two sections were then sutured to the peritoneal cavity and close to the ovaries with a non-absorbable suture 5/0. Layered closure of the abdominal wall and resuscitation of the rats were also performed. After 14 days, the rats were subjected to euthanasia for the surgical investigation to detect possible endometriotic foci, which were removed and sent for histological examination [24–31].

The process of experimental induction of intraperitoneal endometriosis (Group C) involved anesthesia with intraperitoneal (i.p.) administration of ketamine and xylazine, midline surgical incision, the opening of the abdominal wall and detection of the uterine horn, a part of which was removed and crushed and then placed-auto transplanted into the peritoneal cavity. Therefore, endometriosis was surgically induced by implanting autologous parts of endometrial tissues in the peritoneal cavity. All surgical interventions were performed in aseptic conditions. Initially, the rats were placed in a supine position; then, a surgical midline incision of approximately 3–5 cm through the skin was performed. Afterwards, two uterine horns were found. The peripheral part of the left uterine horn, 1 cm long, was ligated at the level of the uterotubal junction, and an incision was made at the proximal end. The part was removed and kept in a sterile recipient containing NaCl 0.9% at 37°C. Next, the right endometrial horn was opened with a surgical scalpel and separated into sections that were crushed and transplanted into the peritoneal cavity. Layered closure of the abdominal wall and resuscitation of the rats was also performed. After 14 days, the rats were subjected to euthanasia, and a surgical investigation was performed to find possible endometrial foci, which were removed and sent for histological examination together with the ovaries [24–31].

In the case of Group D, we created a model of extraperitoneal endometriosis. The process followed was similar to the one used in the case of intraperitoneal endometriosis. The only difference was that the parts of the crushed horn were placed in extraperitoneal locations (subcutaneously). After 14 days, the rats were subjected to euthanasia, and a surgical investigation was performed to find endometriotic foci, which were removed and sent for histological examination together with the ovaries [30–33]. The pathologist was blinded to the treatment method.

Our experiment measured:

1. The presence of endometriosis foci in groups B, C, D;
2. The changes in the body weight of the rats (postoperative weight -preoperative weight);
3. The number of luteinized unruptured follicles per rat.

## RESULTS

**Figure 1:**
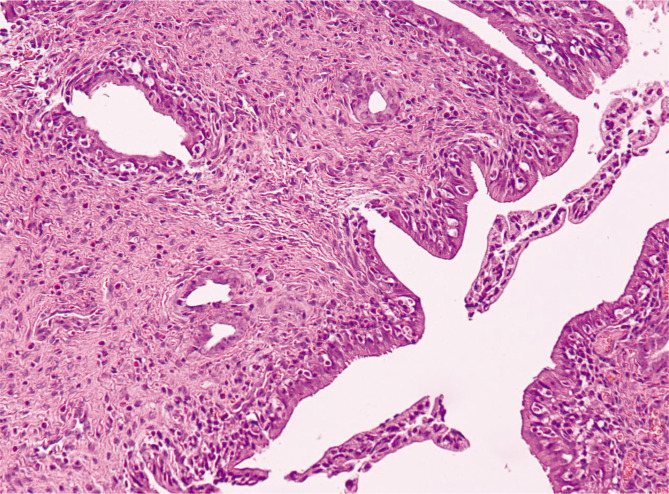
Development of endometriosis in our experimental model of rats.

According to the results of our experiment, all the rats in groups B (6/6) and C (6/6) developed endometriosis, while five out of six rats (5/6) developed endometriosis in group D. The pathologist who performed the histopathological examination of the preparations obtained after killing the rats was not aware of the grouping and classification of the experimental animals. The preparations were fixed in 10% formaldehyde solution, and then paraffin blocks were encapsulated and cut into 5 mm thick sections using a microtome. The presence of endometrial implants was confirmed using hematoxylin-eosin. The histological examination established the diagnosis of endometriosis by identifying the endometrial glandular tissue and the layer (Figure 1). The IBM SPSS Statistics 25 statistical package was used for data analysis. For quantitative variables, the analysis was performed using the t-test for independent samples or the Mann-Whitney U test, depending on the data distribution. Where comparisons of quality variables were required, Pearson's ×2 test was used. The level of statistical significance was set at the value of p≤0.05.

When comparing the changes in the body weight (body weight of the rats at the end of the experiment- body weight of the rats at the beginning of the experiment) of the groups of rats in our experiment, the following were observed: [A (12.8±0.75), B (12.8±0.75), C (12.5±2.42), D (13.00±1.78) with p>0.05)] (Table 1).

**Table 1:** Changes in rats weights from Group A, B, C and D between the preoperative to the postoperative phase.

	Group A	Group B	Group C	Group D	F	P-value
**Change in body weight of rats (g)**	12.8±0.75	12.8±0.75	12.5±2.42	13.00±1.78	0.103	.957

No statistically significant differences were observed in our experiment regarding the changes in the body weight of the rats in groups A, B, C, and D.

Concerning the number of luteinized follicles, rats with endometriosis presented a statistically significant (p<0.05) higher number of luteinized unruptured follicles in relation to the control Group A (Table 2).

**Table 2:** Average number of luteinized unruptured follicles.

Group of rats	Average number of luteinized unruptured follicles
A	0.25±0.51
B	3±0.63
C	2.5±0.55
D	2±0.70

Statistically significant differences (p<0,05) were observed when comparing the average number of luteinized unruptured follicles in group A (0.25±0.51) with groups B (3±0.63) C (2.5±0.55) and D (2±0.70). Consequently, group A presented a lower number of luteinized unruptured follicles in relation to groups B, C and D (Tables 3, 4 and 5).

**Table 3:** Comparison of the number of luteinized unruptured follicles between Group A and B.

	Group A	Group B	U	P-value
**Number of luteinized unruptured follicles**	0.25	3.00	0.000	.002*

**Table 4:** Comparison of the number of luteinized unruptured follicles between Group A and C.

	Group A	Group C	U	P-value
**Number of luteinized unruptured follicles**	0.25	2.5	0.000	.002*

**Table 5:** Comparison of the number of luteinized unruptured follicles between Group A and D.

	Group A	Group D	U	P-value
**Number of luteinized unruptured follicles**	0.25	2.0	0.000	.009*

These results agree with the research which identified that luteinized unruptured follicle syndrome (LUF) was linked to infertility in women [33–35]. Just like women with endometriosis, female rats with surgically induced endometriosis presented an increased incidence of LUF syndrome [36, 37]. Moreover, a study by Yang et al. suggests that the total number of oocytes retrieved was 1.5 fewer in women with surgical insertion of ovarian endometrioma than those without [38–40]. Finally, the research of Stilley 2010 and Moon 1993 showed that rats with endometriosis have fewer follicles [37, 39].

## DISCUSSION

The probability of conception in women with mild endometriosis ranges between 2–10% (15–25% in healthy fertile women) [21, 41]. Based on the literature, extensive fallopian tube-ovarian adhesions or large endometriomas in women with heavy endometriosis prevent ovulation or egg transport to the fallopian tube (mechanical factor) [5, 10, 14, 15, 20–23]. In cases of modest endometriosis, the mechanical factor does not exist. Therefore, other factors are implicated, such as endocrine changes, luteinized unruptured follicle syndrome (LUF), increased level of macrophages, the role of increased levels of prostaglandins, changes in the fallopian tube motility and the fimbriae thereof, egg maturation disorders and immune disorders, such as the increased ratio between ancillary T-cells and suppressor T-cells, the reduced activity of "natural killer" cells and the increased levels of cytokine, [10, 21, 41–44].

Endometriosis has been related to LUF since the 1980s. However, it remains unknown if the mild forms of endometriosis have an immediate effect on the female reproductive system through the negative impact on ovarian histology [45–47].

Studies show an increased level of endometrial prostaglandins in women with endometriosis. The increase in the level of prostaglandins in the peritoneal fluid and the reproductive system results from a) the uterus, b) the ectopic endometrium, c) the increased number of macrophages, and d) the peritoneum itself. Prostaglandins are implicated in dyspareunia and other symptoms of endometriosis such as pathological ovulation, luteal phase disorder and other endocrine changes, which are observed in women with endometriosis. The impact of prostaglandins on the vascular smooth muscle increases the fallopian tube motility and prevents egg transport and implantation resulting in infertility in women with mild endometriosis [10, 48, 49]. Dysfunction of the hypothalamic-pituitary-ovarian axis has also been observed in women with endometriosis, resulting in a luteal phase defect. This defect could be attributed either to an unsuitable feedback mechanism or hyperprolactinemia [24, 50].

*In vitro* (single-cell cultures and co-cultures of more than one type of cells, three-dimensional models and organoids) and *in vivo* models (rodents, rabbits, and non-human primates) [24–26] have been used to understand endometriosis.

The first experiments on endometriosis in primates were conducted in the 1950s. In a simulation attempt of retrograde menstruation, the female monkey cervix was surgically repositioned in such a way as to cause endometrial lesions [25, 26]. The cost of handling these animals was high; therefore, small laboratory animals are currently used to study endometriosis (*i.e*., mice, rats) [26].

Unlike human beings and non-human primates, other animal models do not develop endometriosis spontaneously. Researchers, however, can induce endometriosis in these organisms through the ectopic transplantation of endometrial tissue [45]. Depending on the origin of the tissue used for the induction, the said mouse models can be distinguished into two basic types: homologous and heterologous.

In homologous models, the endometrium is received from the uterus of a related animal, and it is either inserted or dispersed into the peritoneal cavity of a second animal [24]. The reproductive system of homologous models of rodents remains intact and provides an opportunity to study cross-communication between the immune system and the endometriotic cells through the peritoneal microenvironment, which appears to play a major role in humans [25].

Autotransplantation of uterus tissue to ectopic sites of small laboratory animals has been applied multiple times to mice and rats, as well as to rabbits and hamsters. Mice and rats are among the non-human primate models used over the last few years. In these models, the uterus is removed, cut into small pieces, and reimplanted into animals, mainly employing peritoneum sutures [25, 26].

No endometrial separation from the myometrium has been reported in most research cases; therefore, both were implanted. In rats, the uterus tissue is developed in ovoid, fluid-filled, cystic structures consisting of endometrial and myometrial tissue. The said cystic structures increase and stabilize in size after 2 weeks and remain in this state for at least 10 months. In rats, the ectopic sites of the uterus present histological features of human diseases, such as the development of highly vascularized cystic lesions consisting of endometrial stroma and glands [24–26].

In heterologous models, human endometrial parts are received and injected into immunodeficient rats [34]. The heterologous xenograft model uses immunodeficient rats to prevent graft *versus* host reaction that would create a biological state that does not match the chronic inflammatory environment in human endometriosis. Xenotransplantation of human endometrium tissue into immunodeficient rats is usually carried out by injecting a vaccine in the peritoneal cavity, given either subcutaneously or by micro-laparotomy [24–26].

In our experimental animal model, we surgically induced endometriosis by implanting autologous parts of endometrial tissue in the peritoneal cavity. According to our experimental model, endometriosis increases the number of luteinized unruptured follicles. These results support the research that LUF is associated with infertility in women [33–35].

## CONCLUSIONS

Endometriosis is a chronic disease that affects women of reproductive age. Even though subfertility's association with endometriosis is still debatable, clinical observations and various studies support a slight link. Pathogenesis of endometriosis-associated subfertility is not clear thus far, although some data indicate that several factors could affect a patient's fertility.

Mechanisms include mechanical obstruction, such as ovarian and tubal dysfunction, the abnormal peritoneal microenvironment, genetic and epigenetic mechanisms, and immunological traits. It is fundamental to better understand these mechanisms to improve the therapeutic approach. The usual treatment of endometriosis-associated subfertility consists of surgery, ART, and medical treatment. Future researchers should focus on novel non-invasive treatment methods that target specific pathogenic pathways.

The present experimental model aimed to investigate the possible effect of endometriosis on ovarian function by altering follicular maturation and development. According to our experimental model, endometriosis may affect ovarian function by increasing the number of luteinized unruptured follicles.
