# Correction to: The effect of endometriosis on fertility in an animal model

**DOI:** 10.25122/jml-2023-1010

**Published:** 2023-02

**Authors:** Dimitrios Kanellopoulos, Dimitra Karagianni, Vasilios Pergialiotis, Nikolaos Nikiteas, Andreas C Lazaris, Dimitrios Iliopoulos

**Affiliations:** 1 Laboratory of Experimental Surgery and Surgical Research N.S. Christeas, National and Kapodistrian University of Athens, Athens, Greece; 2 1^st^ Department of Pathology, National and Kapodistrian University of Athens, Athens, Greece; 3 2^nd^ Propaedeutic Department of Surgery, National and Kapodistrian University of Athens, Athens, Greece

## Abstract

This corrects the article on pages 1170–1173, vol. 15, PMID: 36415526.

## CORRECTION

In the original publication [1], the term "mice" was mistakenly used instead of "rats" throughout the entire text. The original article has been corrected and all instances of the term "mice" have been replaced with "rats". We apologize for any confusion this error may have caused and appreciate the readers for bringing it to our attention.
